# Solution Structure, Dynamics, and New Antifungal Aspects of the Cysteine-Rich Miniprotein PAFC

**DOI:** 10.3390/ijms22031183

**Published:** 2021-01-25

**Authors:** András Czajlik, Jeanett Holzknecht, László Galgóczy, Liliána Tóth, Péter Poór, Attila Ördög, Györgyi Váradi, Alexander Kühbacher, Attila Borics, Gábor K. Tóth, Florentine Marx, Gyula Batta

**Affiliations:** 1 Department of Organic Chemistry, Faculty of Science and Technology, University of Debrecen, H-4032 Debrecen, Hungary; czajlik.andras@science.unideb.hu; 2 Institute of Molecular Biology, Biocenter, Medical University of Innsbruck, A-6020 Innsbruck, Austria; jeanett.holzknecht@i-med.ac.at (J.H.); alexander.kuehbacher@i-med.ac.at (A.K.); 3 Institute of Plant Biology, Biological Research Centre, Eötvös Loránd Research Network, H-6726 Szeged, Hungary; galgoczi.laszlo@brc.hu (L.G.); toth.liliana@brc.hu (L.T.); 4 Department of Biotechnology, Faculty of Science and Informatics, University of Szeged, H-6726 Szeged, Hungary; 5 Department of Plant Biology, Faculty of Sciences and Informatics, University of Szeged, H-6726 Szeged, Hungary; poorpeti@bio.u-szeged.hu (P.P.); aordog@bio.u-szeged.hu (A.Ö.); 6 Department of Medical Chemistry, Faculty of Medicine, University of Szeged, H-6720 Szeged, Hungary; varadi.gyorgyi@med.u-szeged.hu (G.V.); toth.gabor@med.u-szeged.hu (G.K.T.); 7 Institute of Biochemistry, Biological Research Centre, Eötvös Loránd Research Network, H-6726 Szeged, Hungary; borics.attila@brc.hu; 8 MTA-SZTE Biomimetic Systems Research Group, University of Szeged, Dóm tér 8, H-6720 Szeged, Hungary

**Keywords:** *Penicillium chrysogenum*, antifungal protein PAFC, γ-core motif, solution structure, dynamics, nuclear magnetic resonance, plant protection

## Abstract

The genome of *Penicillium chrysogenum* Q176 contains a gene coding for the 88-amino-acid (aa)-long glycine- and cysteine-rich *P. chrysogenum* antifungal protein C (PAFC). After maturation, the secreted antifungal miniprotein (MP) comprises 64 aa and shares 80% aa identity with the bubble protein (BP) from *Penicillium brevicompactum*, which has a published X-ray structure. Our team expressed isotope (^15^N, ^13^C)-labeled, recombinant PAFC in high yields, which allowed us to determine the solution structure and molecular dynamics by nuclear magnetic resonance (NMR) experiments. The primary structure of PAFC is dominated by 14 glycines, and therefore, whether the four disulfide bonds can stabilize the fold is challenging. Indeed, unlike the few published solution structures of other antifungal MPs from filamentous ascomycetes, the NMR data indicate that PAFC has shorter secondary structure elements and lacks the typical β-barrel structure, though it has a positively charged cavity and a hydrophobic core around the disulfide bonds. Some parts within the two putative γ-core motifs exhibited enhanced dynamics according to a new disorder index presentation of ^15^N-NMR relaxation data. Furthermore, we also provided a more detailed insight into the antifungal spectrum of PAFC, with specific emphasis on fungal plant pathogens. Our results suggest that PAFC could be an effective candidate for the development of new antifungal strategies in agriculture.

## 1. Introduction

Fungal infections of humans, animals, and plants have a severe impact on global health and jeopardize the food supply and wildlife biodiversity [1]. A major challenge in combating fungal diseases is the limited number of targets for effective antifungal therapy that is tolerated by the host. Therefore, the development of antifungal drug resistance poses a dangerous and fast-evolving risk, which strongly demands the search for new antifungal compounds with novel mechanisms of action and fungal-specific unique targets. Very promising candidates [2,3,4,5] for new antifungal strategies are represented by small, cysteine-rich, and cationic proteins from diverse organisms [6]. Filamentous ascomycetes are a rich source of secreted miniproteins (MPs) that exhibit high and exclusive efficacy against the growth of human, animal, and plant pathogenic fungi [7,8,9], and the first evidence promises antiviral efficacy as well [10]. These small and positively charged MPs have disulfide-stabilized β-barrel folds that render them tolerant toward high ion concentrations, extreme temperature, and proteolytic degradation [8,9,10,11,12,13,14]. Since no cytotoxic effects in mammalian cells have been described in vitro and in vivo so far [9,11,12,13], they are considered promising candidates for alternative antifungal strategies in clinics, veterinary science, agriculture, and food production.

*Penicillium chrysogenum* is unique among filamentous ascomycetes as its genome contains three genes coding for cysteine-rich, cationic MPs with reported antifungal activity: the *P. chrysogenum* antifungal protein (PAF), *P. chrysogenum* antifungal protein B (PAFB), and *P. chrysogenum* antifungal protein C (PAFC) [9,14]. It is known that *P. chrysogenum* MPs enter sensitive fungal cells without disrupting the plasma membrane, but they accumulate in the cytoplasm, induce the generation of intracellular reactive oxygen species (ROS), and ultimately trigger cell death [9,10,14,15]. The extensively studied PAF has a complex antifungal mode of action that is linked to cell signaling involving heterotrimeric G-protein [16] and protein kinase A [17], cation channels [18,19], and glucosylceramide synthesis [20] and is regulated by a specific protein motif that structurally resembles the Greek letter gamma (γ) [21]. This common, so-called γ-core motif with the consensus sequence GXCX_3-9_C (dextromeric form) or CX_3-9_CXGX_1-3_/CX_3-9_GXCX_1-3_ (levomeric forms) is found in many cysteine-stabilized MPs originating from organisms of diverse kingdoms [22,23].

**Figure 1:**
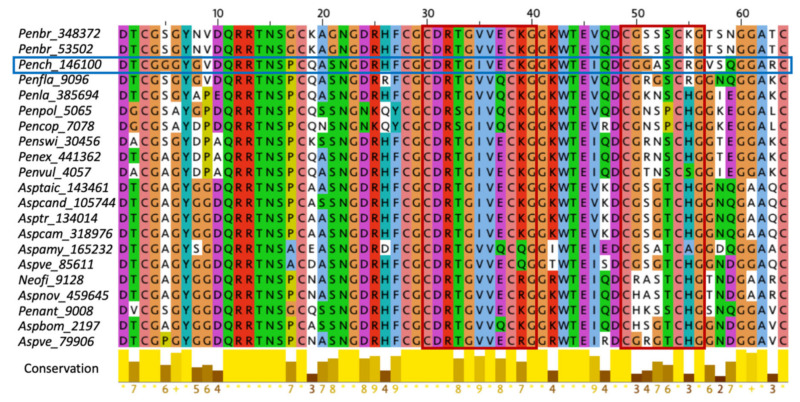
ClustalW multiple alignment of the mature, *P. brevicompactum* “bubble protein” BP-clade-specific MPs of Eurotiomycetes [21]. *P. chrysogenum* PAFC (Pench_146100) is framed in blue. The two conserved γ-core motifs found in the members of this clade are framed in red. The abbreviations of the full species names and the protein accession number are indicated: *Penbr* (*P. brevicompactum*), *Pench* (*P. chrysogenum*), *Penfla* (*Penicillium falvigenum*) *Penla* (*Penicillium lanosocoeruleum*), *Penpol* (*Penicillium polonicum*), *Pencop* (*Penicillium coprophilum*), *Penswi* (*Penicillium swiecickii*), *Penex* (*Penicillium expansum*), *Penvul* (*Penicillium vulpinum*), *Asptaic* (*Aspergillus taichungensis*), *Aspcand* (*Aspergillus candidus*), *Asptr* (*Aspergillus triticus*), *Aspcam* (*Aspergillus campestris*), *Aspeamy* (Aspergillus amylovorus), *Aspve* (*Aspergillus versicolor*), *Neofi* (*Neosartorya fischeri*), *Aspnov* (*Aspergillus novofumigatus*), *Penant* (*Penicillium antarticum*), and *Aspbom* (*Aspergillus bombycis*). The color coding of aa was applied according to ClustalX. The conservation between the respective sequences based on the ClustalW2 Multiple Sequence Alignment tool [26] is indicated at the bottom.

The mature PAFC [14] is a unique example of antifungal MPs, since it has a molecular mass of 6.63 kDa and is slightly longer (64 amino acids (aa)) than the mature PAF (55 aa; 6.25 kDa) and PAFB (58 aa; 6.55 kDa) [10,11]. It shows 80% aa identity with the bubble protein (BP) of *Penicillium brevicompactum* [24] and 83% identity with the *Penicillium expansum* antifungal protein C (PeAfpC) [25] (Figure 1). Therefore, it phylogenetically belongs to the BP-clade of ascomycetous MPs, which is distinct from the PAF-clade that comprises the PAFC-related MPs PAF and PAFB [21]. PAFC contains an unusually high number of glycines (14) and has a net charge of +3.6 ± 0.1 at pH 4.5, as calculated considering the disulfide bonds, using in-house-written MATLAB code. This is due to a smaller number of cationic residues (6 arginines, 2 lysines, and 1 histidine) compared to PAF (13 lysines; net charge +6.7 ± 0.1; pH 4.5) and PAFB (8 lysines, 2 arginines, and 4 histidines; net charge +11.5 ± 0.2; pH 4.5). Furthermore, PAFC has two putative levomeric γ-core motifs (CX_3-9_CXGX_1-3_). One of them—positioned in the center of PAFC (CDRTGIVECKG)—is highly conserved among the MPs of the BP-clade, while the second, shorter one with lower homology resides near the C-terminus (CGGASCRG) (Figure 1) [14].

In previous studies, we acquired nuclear magnetic resonance (NMR) structure data of MPs of the filamentous ascomycetes *Penicillium chrysogenum* (PAF, 2mhv) [19,27], short form of PAFB, (sfPAFB, 2nc2) [9,10], and *Neosartorya fischeri* antifungal protein (NFAP, 5oqs) [28,29,30] that support a better understanding of the protein function and foster the improvement in antifungal efficacy by intentional modifications (protein engineering) [21].

So far, information about the three-dimensional structural data of MPs of the BP-clade is limited, as an X-ray structure was published only for the *P. brevicompactum* BP [24] and an *in silico* structure prediction exists for PAFC [14]. As the aa sequence of PAFC shows special features, our emphasis in this study was to experimentally verify that this MP has a folded structure, since the glycine-rich sequence requires four disulfide bonds to achieve folding. By acquiring the solution structure of PAFC, we disclosed its structural similarity to and differences from other antifungal MPs from ascomycetes. An overall low positive net charge, a short helix besides the five β-strands, and a positively charged cavity distinguish PAFC from the other two *P. chrysogenum* antifungal proteins PAF and PAFB. NMR relaxation-based dynamics was used to verify the monomeric and mostly rigid structure of PAFC. Dynamical peculiarities of the γ-core motif are important observations, since these regions are reported to contribute to the antimicrobial impact and spectrum of defensins and cationic MPs [22]. Finally, we provide an extended insight into the antimicrobial potential of PAFC and prove its specific antifungal but not antibacterial efficacy. As a proof-of-principle, we show the applicability of PAFC as a bio-fungicide in agriculture, as it protects tomato plant leaves against infection by the necrotrophic plant pathogen *Botrytis cinerea*.

## 2. Results

### 2.1. Characterization of Structural and Dynamical Properties of PAFC

Solution NMR spectroscopy was employed with the ^15^N- and ^13^C-labeled recombinant PAFC. All amide backbone and side-chain groups could be identified and assigned in the ^1^H-^15^N heteronuclear single quantum correlation (HSQC) spectrum due to the high dispersion of amide (NH) signals (Supplementary Figure S1). This fingerprint clearly showed that the protein adopts a well-folded structure. Many NH signals are split by high ^3^J (HN, HA) couplings, typical for β-strands. The large majority (97.1%, 90.6%, and 85.1%) of the ^15^N, ^13^C (all carbons, without aromatics), and ^1^H chemical shifts were successfully assigned. More than the half of the nuclear Overhauser effects (NOEs) proved to be either medium- (12.0%) or long-range (43.9%) interactions, which indicated a β-structure for PAFC. The C_β_^13^C chemical shift values of all cysteines were typical for disulfide bridges [31], as expected. PAFC has the same abcabdcd-type disulfide bond pattern as BP, connecting 3-30, 18-38, 28-54, and 49-64 cysteines. This assumption was firmly supported by the structure calculations. When no disulfide bond constraints were added to the NOE constraints, nearly identical structures were obtained as with the disulfide-constrained cases. The high number of distance (14.3 NOE/residue) and backbone torsional angle constraints allowed us to calculate a high-quality NMR structure with a low (0.55 ± 0.06 Å) heavy-atom root-mean-square deviation (RMSD) value in the final model. The three-dimensional structure and chemical shift assignments of PAFC were deposited to the Protein Data Bank (pdb code 6TRM) and the Biological Magnetic Resonance Data Bank (BMRB entry no. 34468), respectively. According to official pdb validation analysis, only Cys3 was an outlier for Ramachandran statistics (Supplementary Table S1) of backbone dihedral angles, and according to our records no NOE violations were found.

**Figure 2:**
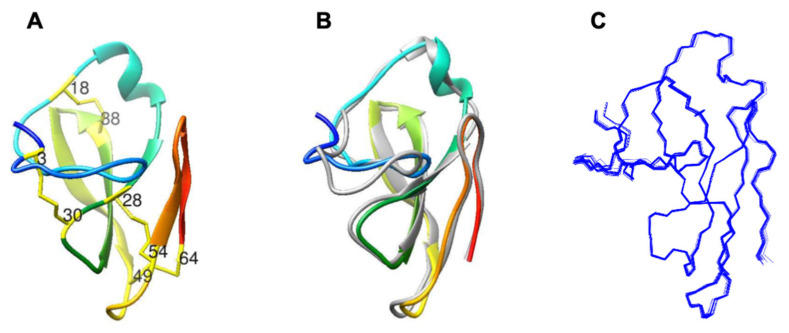
Comparison of the structures of *Penicillium* spp. MPs. (**A**) The NMR solution structure of the PAFC (pdb code 6TRM). The position of the Cys residues is indicated, and the disulfide bonds are labeled as yellow sticks. (**B**) Superimposed structures of the PAFC (pdb code 6TRM) and the *P. brevicompactum* BP (gray color; pdb code: 1UOY) using Chimera visualization software [33]. (**C**) Backbone NMR conformational ensemble of 20 structures of PAFC.

The three-dimensional structure of PAFC (Figure 2A) shows the characteristic fold of the BP-clade as aligned with BP (Figure 2B), with an RMSD deviation of 1.01 Å between the two structures. The N-terminal part of the protein (1–25) does not form strict secondary structure elements. Though the random coil index (RCI) [32] derived from NMR chemical shifts was well below the critical 0.2 value, a peak value of 0.15 close to the N-terminus (around Gly8) is a sign of disorder. Also, according to the lower number of NOEs at the N-terminus, the conformations of the first seven residues are less well-defined if compared to the rest of PAFC. The next region (aa 8–19) adopts a mostly extended structure, interrupted by bend and turn-like motives (aa 9–10, aa 14–15). It is followed by a short and irregular 3_10_-helix (Ala20–Asp24). In contrast, the C-terminal part of the protein forms a β-strand structure with two antiparallel β-sheets. The first one consists of three (β1, β2, and β3) and the latter one of two (β4, β5) β-strands. The positions of strands are His26–Cys28 for β1, Gly34–Lys39 for β2, Lys42–Asp48 for β3, Arg55–Val57 for β4, and Gly60–Arg63 for β5. The first, longer β-sheet is somewhat right-handed twisted, similarly to that of BP. Although the second one proved to be more irregular, still all the possible H-bonds were experimentally identified in this region (Table 1).

**Figure 3:**
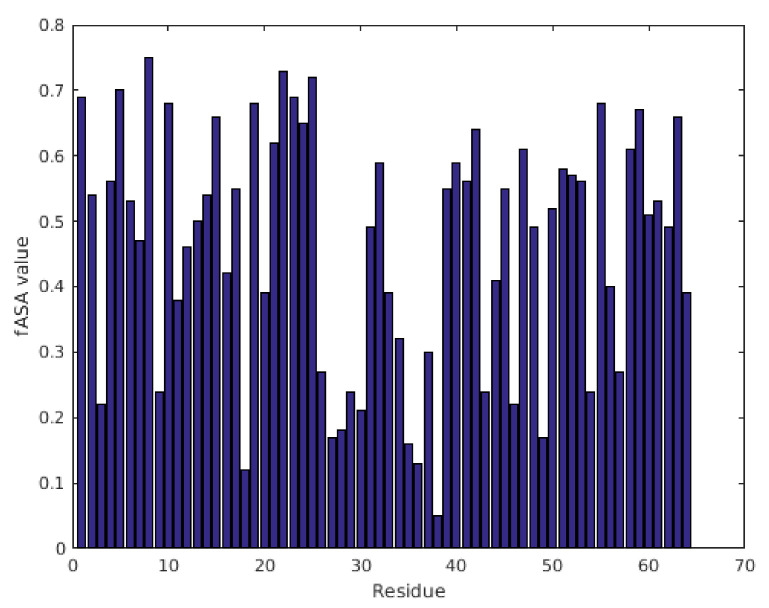
fASA values of PAFC calculated from ^13^C chemical shift data show residue-by-residue hydrophobicity of the structure. Residues with fASA values below 0.25 are considered strongly buried, while fASA values above 0.75 indicate exposure to solvent.

More detailed examination of the β-strands revealed the amphipathic nature of β1, β2-, β4-, and β5-strands. Interestingly, both C-terminal strands are partially positively charged, and they do not contain any acidic residues. In contrast, β3 consists of several charged aa, and it is rather hydrophilic based on the aa sequence, especially compared to other parts of the protein. The fractional associated solvent area (fASA) parameters, as obtained from ^13^C chemical shifts [35], report on the buried parts of the structure (Figure 3). Low fASA values are characteristic for buried residues. In PAFC, most minima are centered around the cysteines, thereby proving the existence of the usual disulfide-protected hydrophobic inner core of antifungal MPs [10,30].

Four small loops are situated between the β-strands. Three of them form β-turns (Cys30–Asp31, Gly40–Gly41, Ser58–Gln59), and the fourth, located between the two sheets, shows irregular γ-turn motives (at Gly50 and Ala52). The secondary structural analysis described above is consistent with that based on the Dictionary of Secondary Structures of Protein (DSSP) method [36]. PAFC, as a member of the BP-clade proteins, contains four disulfide bridges. While the first two (3–30, 18–38) connect the N-terminal region to the first β-pleated sheet, the others (28–54, 49–64) are located between the two sheets. This arrangement strongly stabilizes the tertiary structure of the protein, and this stability is further supported by several H-bonds formed between the N-terminal extended part and the β-sheets in the order of NH donor and CO acceptor sites (12Arg–28Cys, 28Cys–10Asp, 15Asn–43Trp, and 43Trp–16Ser). In PAFC, the side chains of Asp31, Glu45, and Asp48 are close to each other and form a small negatively charged surface region with the hydrophilic residues Thr33 and Ser53. Interestingly, the most basic side chains face outside and do not interact with one another or any acidic residues. One possible salt bridge can be identified between the side chains of Glu37 and His26, and only two basic arginines (32 and 55) are close to each other. Though the aromatic side chains Trp43 and Phe27 interact with each other to some extent, no significant aromatic core is present in PAFC and only Arg12 is in their vicinity. In contrast, a small hydrophobic core with Ile35 in the center is found near the C-terminal disulfide bonds, in agreement with low fASA values. 

**Figure 4:**
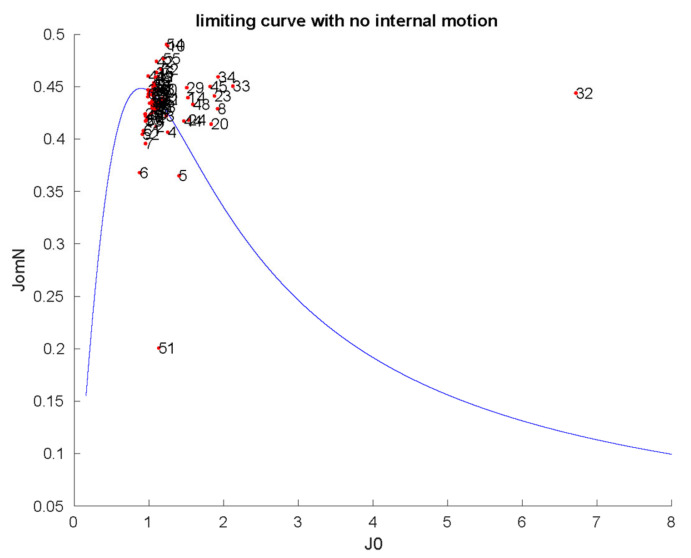
Reduced spectral density mapping of NH mobilities in PAFC (298 K). The limiting continuous curve represents the absence of internal motion, as calculated by τ_c_ = 3.14 ns global correlation time. Spectral densities (the strength of fluctuating radiofrequency fields from molecular rotational diffusion) at ^15^N frequency shown as a function of such fields close to zero frequency (slow-motion regime) [40]. JomN on the vertical axis shows the spectral densities at ^15^N frequency, while J0 on the horizontal axis is proportional to spectral densities close to zero frequency. The units of the two axes should be understood as 10^−9^ (s rad^−1^).

For studying the dynamical behavior of PAFC, ^15^N (70.96 MHz) NMR relaxation (T_1_, T_2_, NOE) measurements [37] were performed and evaluated using the Lipari–Szabo model-free method [38]. The 3.14 ± 0.36 ns global correlation time (*τ*_c_) is similar to other monomeric antifungal MPs (PAF, NFAP). Details of relaxation analysis are provided in Table S2. The average S^2^ order parameter was calculated as S^2^ = 0.76 ± 0.11, which shows that PAFC has slightly higher flexibility on the ps–ns timescale if compared to PAF or NFAP [19,30]. To some surprise, the S^2^ values of even the N-terminal region proved to be similar to those of the β-strands (Supplementary Figure S2). Nevertheless, some residues are somewhat less rigid, including Gly6, Asn15, Glu37, Gly41, Gln47, Gly60, Gly61, and, especially, Gly51 and Ala52. Analysis of the R_1_*R_2_ relaxation rate combinations [39] showed that Arg32 and possibly Gly51 must be affected by chemical exchange (Supplementary Figure S3). The conventional reduced spectral density mapping approach (RSDM) [40] also suggests enhanced motions around Arg32 and Gly51 and to a lesser extent at Gly6, Tyr7, Gly8, Gly23, Thr33, Gly34, and E45 (Figure 4). Thus, the end of the first loop plus the β2-strand and the third loop are somewhat more flexible with higher mobility on the ps–ns timescale. However, Carr-Purcell-Meiboom-Gill relaxation dispersion (CPMG-RD) experiments could not detect mobility on a much slower (μs–ms) timescale (data not shown).

**Figure 5:**
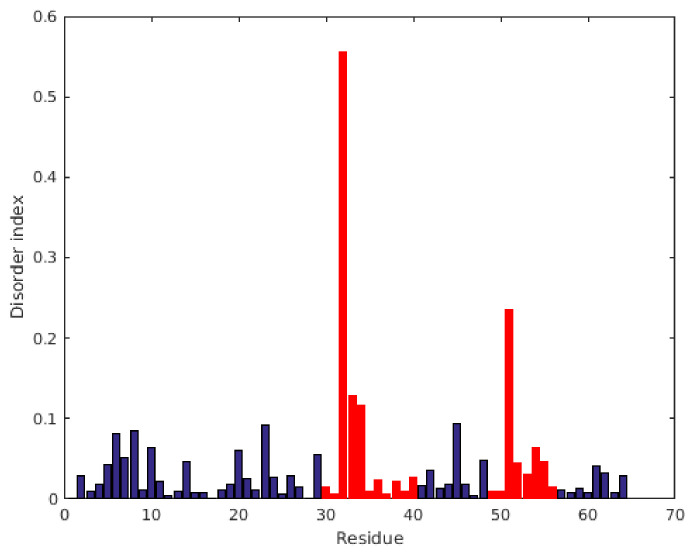
Disorder index (DI), as obtained directly from reduced spectral density mapping of ^15^N NMR relaxation data. The DI is just derived from Figure 4 by calculating the geometrical (shortest) distances of the points from the solid (limiting) curve. The γ-core regions are labeled as red bars, while other residues are blue.

For better visualization of the same ^15^N relaxation data, we introduced a so-called disorder index, where the geometric distance from the rigid-body limiting curve of Figure 4 is displayed as a function of the residue number. Figure 5 clearly shows that extreme dynamics appears in the two γ-core regions between residues 30–40 and 49–56. Finally, we compared the RSDM, R_1_*R_2_, and model-free (MF) methods. Using the first two methods, we found that slow exchange must persist in region 32–34, which is not reflected in MF S^2^ values. On the other hand, the S^2^ values are low at Gly51 (0.39) and Ala52 (0.58), suggesting enhanced dynamics here at the ps–ns timescale. This fast timescale dynamics was not sensed by the R_1_*R_2_ method. In contrast, the more general RSDM methods displayed enhanced dynamics in both regions, independent of the timescale. Interestingly, ^15^N CPMG-RD T_2_ relaxation dispersion experiments were insensitive in detecting dynamics at intermediate timescales (μs–ms range).

### 2.2. Secondary Structure and Thermal Stability Analysis of PAFC Using Electronic Circular Dichroism Spectroscopy

**Figure 6:**
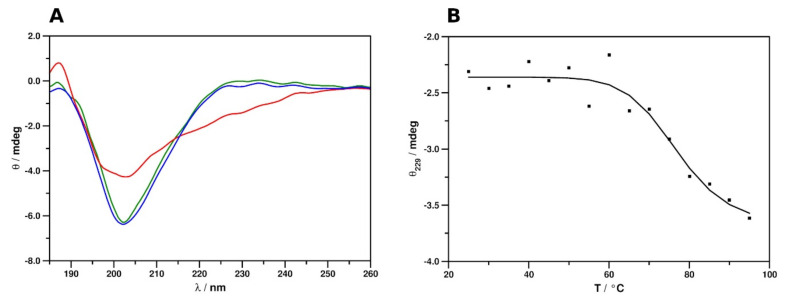
ECD spectra and thermal unfolding curve of PAFC. (**A**) Spectra in the 185–260 nm region were measured at 25 °C (green), 95 °C (red), and again 25 °C (blue) after refolding. (**B**) Thermal unfolding of PAFC, followed by ECD spectra at 229 nm. *T*_m_ = 77 °C.

The electronic circular dichroism (ECD) spectrum of PAFC at 25 °C showed markedly different features to those observed previously for the related proteins (Figure 6A) PAF [41], PdAfpB from *Penicillium digitatum* [42], and NFAP and NFAP2 from *Neosartorya fischeri* [43,44]. The spectrum was characterized by a broad, intensive minimum at 202 nm, which could indicate the presence of multiple structural elements with the predominance of atypical, non-canonical secondary structures. A contribution from a very low intensity maximum, centering around 229 nm, was revealed by comparison to the spectrum acquired at 95 °C. This low-intensity maximum indicated the presence of disulfide bridges with uniform geometries. This suggested, in accordance with the results provided by NMR spectroscopic analysis, that the predominance of highly dynamic disordered structures could be ruled out and that the protein adopts a stable tertiary structure in aqueous solution. Monitoring the change of spectral intensity at 229 nm at elevated temperatures yielded an inverse sigmoid-type unfolding curve (Figure 6B), similar to those acquired previously for other cysteine-rich, cationic, antifungal MPs [41,42,43,44]. Fitting the data points with an inverse sigmoid function (*R*^2^ = 0.936) appointed *x* = 76.83653 as the inflection point of the fitted curve. Therefore, the melting point of the protein structure (*T*_m_) could be estimated to be approx. 77 °C. This indicates remarkable thermal stability, similar to that of related proteins. The spectrum measured again at 25 °C after heat treatment (Figure 6A) confirmed that the unfolding of PAFC is entirely reversible and corroborates the preservation of anti-*Candida* activity after heat treatment, as reported recently [14].

Spectral deconvolution data and the estimation of secondary structural components are included in Table 2. While the contributions of helical and unordered segments are slightly overestimated by ECD spectra at the expense of β-strands and turn structures, there is an acceptable agreement between ECD and NMR data, especially when considering the limited site specificity of the former.

### 2.3. Microbial Growth Inhibitory Activity of PAFC

Recombinant, unlabeled PAFC was tested for antimicrobial activity, and the inhibitory concentration that reduces growth by ≥90% (IC_90_) was determined (Table 3). The microorganisms tested in broth microdilution assays comprised Gram-positive (*Bacillus subtilis)* and Gram-negative (*Escherichia coli*) bacteria and filamentous fungi (the PAFC producer *P. chrysogenum*, the model organisms *Aspergillus nidulans, Aspergillus niger* and *Neurospora crassa*, the opportunistic human pathogen *Aspergillus fumigatus*, and the dermatophytes *Microsporum gypseum* and *Trichophyton rubrum*). Filamentous fungi showed high tolerance (*A. nidulans*) or resistance (*P. chrysogenum*, *A. fumigatus*, and *A. niger*) to PAFC in the tested concentration range. Interestingly, *Microsporum gypseum* and *Trichophyton rubrum* proved to be the most sensitive ones (IC_90_ 5–7 µM). No growth inhibitory activity was observed for bacteria at concentrations tested up to 50 μM.

### 2.4. The Role of the γ-Core Motif in the Antifungal Activity of PAFC

**Figure 7:**
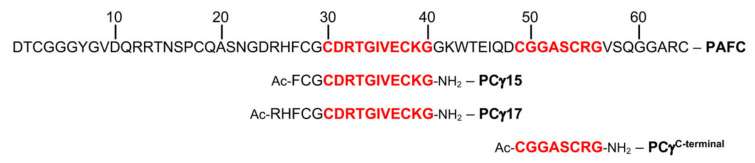
The primary structure of PAFC and the derived synthetic γ-core peptides. The aa sequence of PAFC is indicated by a one-letter code. The predicted levomeric γ-core motifs in the center and the C-terminus are highlighted in red. Below, sequences of the N-terminally acetylated (Ac-) synthetic peptides PCγ15, PCγ17, and PCγ^C-terminal^ are indicated.

In a previous study, we showed that the peptide spanning the dextromeric γ-core motif of PAF effectively inhibits fungal growth by its own and the efficacy can be increased by modulating its physicochemical properties, i.e., increase in the positive net charge and hydrophilicity [21]. To assess the importance of the two levomeric γ-core motifs for PAFC activity, two peptides spanning the highly conserved motif (CDRTGIVECKG) residing in the protein center and one peptide covering the γ-core (CGGASCRG) at the protein C-terminus were chemically synthesized. The 15-aa-length peptide PCγ15 (FCGCDRTGIVECKGG) comprised the additional residues Phe-Cys-Gly at its N-terminus and showed an almost neutral net charge (−0.2 at pH 7) and a higher grand average of hydropathy (GRAVY) value (−0.087) than the full-length protein (charge +0.6 at pH 7, GRAVY −0.767) (Table 4). PCγ17, a 17-aa-long peptide (RHFCGCDRTGIVECKGG) included the two additional basic residues Arg and His at its N-terminus. It therefore exhibited a higher positive net charge (+1.1 at pH 7) than PAFC and PCγ15 and a GRAVY value of −0.376, rendering this peptide more hydrophilic than PCγ15. Lastly, to determine the role of the putative C-terminal γ-core, the 8-aa-long peptide PCγ^C-terminal^ (CGGASCRG) was synthesized, having a similar positive net charge as PAFC (+0.8 at pH 7) but showing the highest GRAVY value of +0.038 (Figure 7 and Table 4).

Determination of the antifungal potential of the peptides was performed by testing their growth inhibitory activity on the human pathogenic yeast *Candida albicans* and the filamentous model fungus *N. crassa* up to concentrations of 100 µM. Interestingly, Pγ15 and PCγ^C-terminal^ did not inhibit fungal growth in the tested concentration range. PCγ17, however, was effective against *N. crassa* at an IC_90_ of 25 µM but not against *C. albicans*, which is sensitive to the full-length PAFC, as recently reported [14]. The positive net charge and hydrophobicity are important determinants of the antifungal potential of γ-core peptides and their analogues [9,21,44,45,46]. Our data indicated that the predicted canonical PAFC γ-core motives are not functional per se as long as the positive charge is low and the GRAVY value high. The increase in the net charge and the hydrophilicity of the peptide enhance the species-specific antifungal activity. However, the efficacy is still ten-fold lower than that of the full-length protein. This let us conclude that other protein parts are necessary to execute to full antifungal activity.

### 2.5. Effect of PAFC on Medicago truncatula Seedlings

**Figure 8:**
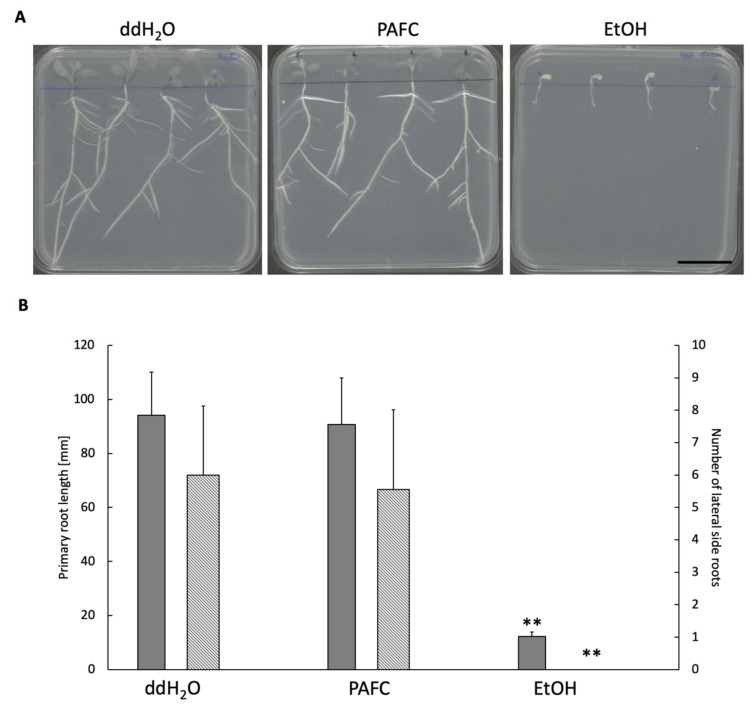
Vegetative growth and root development of *Medicago truncatula* A-17. (**A**) Morphology of plant seedlings and (**B**) primary root length (gray bars) and number of lateral roots (hatched bars) after daily treatment with 1 mg mL^−1^ of PAFC for 10 days at 25 °C under continuous illumination (1200 lux) compared to the ddH_2_O- and 70% (*v*/*v*) ethanol-treated controls, respectively. Scale bar, 30 mm. Significant difference in (**B**) was evaluated with the two-sample *t*-test and is indicated with ** (*p* < 0.005).

Recently, we reported on the efficacy of PAFC to inhibit the growth of one of the most prevalent human pathogens, the yeast *C. albicans*, which renders this MP a promising candidate for the development of new drugs for the treatment of skin and cutaneous fungal infections [14]. In this study, we provide a proof-of-principle that PAFC also protects plants from fungal infection. To consider this MP as a biocontrol agent in agriculture, it is of utmost importance to first exclude any harmful effect on the growth and development of plants. The small and fast-growing legume *Medicago truncatula* is an appropriate model plant to study the harmful effects of pesticides and antifungal compounds like MPs [47]. Thus, *M. truncatula* A-17 seedlings grown on water agar in Petri dishes were daily treated with 1 mg mL^−1^ of PAFC to evaluate the primary root length and the number of lateral roots. No harmful effects were detected after 10 days of incubation between the PAFC-exposed samples compared to the ddH_2_O-treated negative control, whereas significant changes in the root morphology were observed with the positive control, which was daily treated with 70% (*v*/*v*) ethanol (Figure 8).

### 2.6. Protection of Tomato Plant Leaves from Botrytis cinerea Infection

To prove that PAFC can protect plants from fungal infection, we applied a test system that uses the necrotrophic surface plant pathogen *Botrytis cinerea* that infects tomato plant leaves. To approximate more natural conditions, we used the agar diffusion assay instead of the broth microdilution assay to investigate the antifungal potential of PAFC on this filamentous plant pathogen growing on solid surfaces. PAFC was applied into the wells of agar plates in a concentration range of 37–151 μM (0.25–1 mg mL^−1^). The determination of the diameters of the inhibition zones around the wells correlated with the PAFC concentration, whereas no growth inhibition could be detected around the ddH_2_O control well (Table 5).

Microscopy revealed disintegrated conidia and a very low number of germlings in the inhibitory zone around the wells in which 1 mg mL^−1^ of PAFC had been applied, while healthy, well-developed hyphae were found around the control well containing ddH_2_O (Supplementary Figure S4).

**Figure 9:**
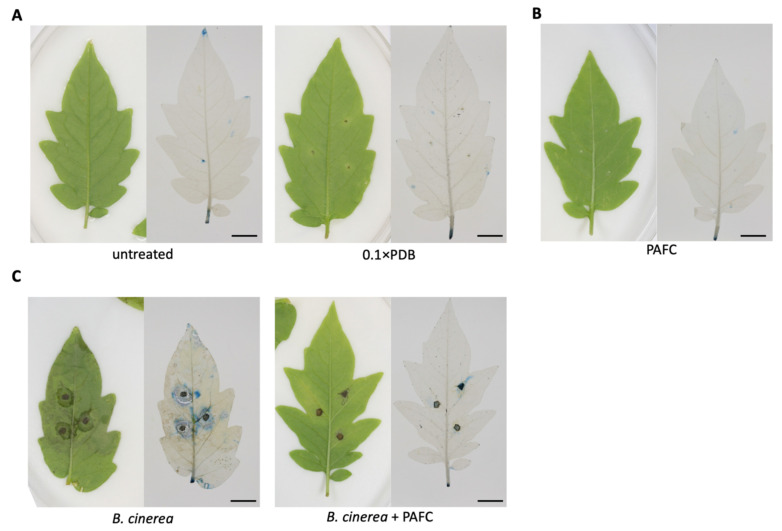
PAFC tolerance of tomato plant leaves and protective effect of PAFC against *B. cinerea* infection. The phenotype of leaves is shown in the left panels and damage evaluation with Evan’s blue staining in the right panels. (**A**) Leaves were either left untreated or treated with 10 µL of 0.1 × PDB (negative controls). (**B**) For toxicity testing, leaves were treated with 10 µL of PAFC (1 mg mL^−1^). (**C**) Plant protection efficacy was evaluated with leaves infected with 10 µL of *B. cinerea* conidia (10^7^ conidia mL^−1^) (infection control) and conidia mixed with PAFC (1 mg mL^−1^). Plants were then further incubated for four days at 23 °C under photoperiodic day/night simulation (12/12 h with or without illumination at 1200 lux) and then harvested for staining. Scale bar, 10 mm.

Next, we infected tomato plant leaves by applying *B. cinerea* spores alone (infection control) or together with 1 mg mL^−1^ of PAFC onto the abaxial leaf epidermis at three points per leaf between the lateral veins and analyzed necrosis development around the application sites after four days of incubation (Figure 9). Leaves similarly treated with PAFC alone (PAFC toxicity control), treated with 0.1 × PDB (medium toxicity control), or left untreated served as negative controls. Evan’s blue staining was performed to detect necrotic zones and revealed no tissue damage in any of the controls (Figure 9A,B), corroborating the tolerance of PAFC by tomato plant leaves. Instead, considerable necrotic tissue around the side of *B. cinerea* application became visible in the infection control (Figure 9C, left side). The presence of PAFC in the infective inoculum, however, significantly reduced necrotic areas on the leaves (Figure 9C, right side). These data clearly indicated that PAFC effectively inhibits *B. cinerea* growth on the plant leaf surface.

## 3. Discussion

**Figure 10:**
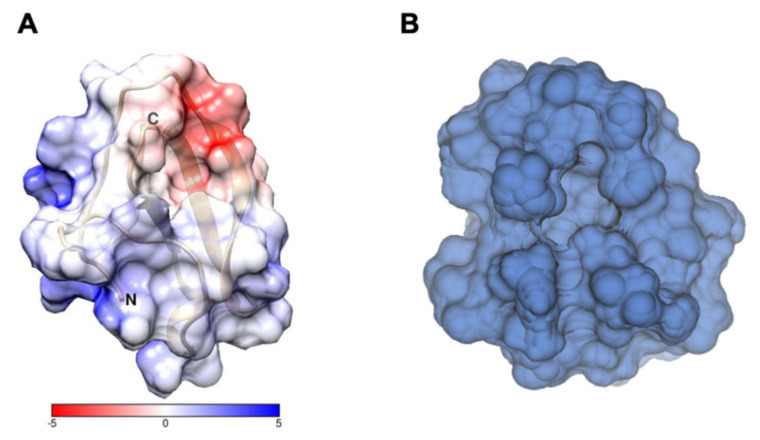
(**A**) Electrostatic potential surface of PAFC, as calculated [48] from the 6TRM structure deposited in pdb. Red means negative, and blue means positive surfaces. The scale is in kJ/mol/e. (**B**) Front view of cavity B of PAFC, formed predominantly by residues 3, 12, 13, 14, 28, 29, 30, 31, 33, 34, 35, 36, 44, 45, and 48.

To unravel the PAFC solution structure at the atomic level, a detailed NMR study was undertaken. The PAFC protein belongs to the BP-clade of ascomycetous MPs. The three-dimensional structure of only one member thereof, the BP from *P. brevicompactum,* was determined using X-ray crystallography [24]. For PAFC, homology-based *in silico* structure prediction was performed earlier [14], which suggested the same fold (RMSD = 0.46 Å) as BP. Therefore, only the two experimental atomic-level structures were compared here. Since PAFC and BP have high sequence identity (80%), it is not surprising that their folds are very similar. They form a long N-terminal region with mostly extended and a short helix-like conformation and two antiparallel β-pleated sheet systems at the C-terminus. In both cases, four disulfide bridges are formed with the same abcabdcd pattern. In fact, the two proteins resemble each other so much that the backbone RMSD is only 1.01 Å. The main difference between them is that the N-terminus between residues 1–7 is slightly less ordered in PAFC than in BP. The same is true for the C-terminal β-sheet, which is shorter and more irregular in PAFC. Similar to BP, there is a hole in PAFC, which we call cavity A (Supplementary Figure S5), which seems to be a characteristic feature of the members of the BP-clade [24]. The outer margin of this funnel consists of several positively charged residues (Arg12, Arg13, and Arg25), which confer this part an overall basic nature. However, the side chains of these aa do not interact with each other, and no strong positively charged patch is present in the protein. The inner base of the funnel is formed by the aromatic Phe27 and Trp43 and in part by the charged Arg12. It is important to note that the cavity A structure is similar to that found in BP and it is formed by highly conserved residues [24]. This suggests that it may play an important functional role for BP-clade proteins. Interestingly, a second hole (cavity B, Figure 10B) can also be observed in PAFC. It is more hydrophilic and predominantly formed by the negatively charged Asp31, Glu45, and Asp48 as well as the basic Arg12 main chain, Arg13 and Arg32, and the hydrophilic residues Thr14, Thr33, and Thr44. The negatively charged region identified in BP was searched also in PAFC. Although it exists in PAFC as well, it is smaller there (Asp31, Asp48, and Glu45) and does not include the C-terminal region [14]. The electrostatic surface potential of PAFC is shown in Figure 10A, as calculated from the pdb-deposited 6TRM structure.

The dynamical properties of the PAFC were studied and compared with other antimicrobial MPs, such as PAF, NFAP, and PAFB, possessing β-barrel structures [10,19,30]. According to this study, the folded PAFC is mostly rigid; however, the N-terminal is more dynamical, and the C-terminal part of the first loop combined with the β2-strand and the long loop between the two β-sheets show some internal motion. Surprisingly, the second and third regions totally overlap the two γ-core motifs identified in PAFC. Although the antifungal MPs of the PAF-clade also contain one γ-core motif, these proteins do not exhibit extreme dynamic regions [10,19,30]. Interestingly, cavity B in PAFC involves the most dynamic part of the first γ-core region. So the flexible regions found in both γ-core regions of PAFC might have a potential role for ligand binding, allowing the conformational selection mode [49] for target molecule recognition.

The evolutionary conserved γ-core motif, which is present in two levomeric forms in PAFC, is an important structural and/or functional component found in disulfide-stabilized proteins and peptides from organisms belonging to all kingdoms [22,23]. In MPs of filamentous ascomycetes, this motif localizes in conserved positions, being negatively charged, positively charged, or neutral [21]. Interestingly, the three members of the BP-clade (BP, PAFC, and PeAfpC) contain two levomeric motifs, the one in the central position being neutral or slightly negatively charged and a more positively charged one at the C-terminus [21]. It is the first time that the impact of the γ-core motif of BP-clade MPs on their function was investigated using synthetic peptides that span these regions. While the γ-core was shown to significantly contribute to the antifungal activity of PAF [21], this role could not be assigned yet to the respective motives in PAFC. Neither PCγ15 nor PCγ^C-terminal^ showed any efficacy against the growth of *N. crassa* and *C. albicans*. Instead, only the N-terminally extended PCγ17 having a higher positive net charge and being more hydrophilic exhibited a detectable IC_90_ against the filamentous model fungus but not against the yeast cells. This indicates that physicochemical features such as a positive net charge and hydrophilicity of the synthetic peptide play an important role in its species-specific function, but not the motif as such, and structural and functional support from other parts of the protein is required for full MP activity. This is consistent with studies performed with synthetic peptides comprising the dextromeric γ-core motif of the *N. fischeri* antifungal protein NFAP2 [44] and the *P. digitatum* antifungal protein AfpB [42], which had no fungal growth inhibitory activity.

By addressing the functional features in more detail, we provide evidence that PAFC exclusively inhibits the growth of filamentous fungi, as no antibacterial activity could be detected. These results complement the reported anti-*Candida* efficacy of PAFC [14], the anti-yeast activity of the *P. brevicompactum* BP [24], and the fungal growth inhibitory activity of Pc-Arctin from the artic sediment isolate *P. chrysogenum* A096, which is identical to PAFC [50]. Interestingly, PAFC was moderately active against its producer *P. chrysogenum.* Self-inhibition by the production of MPs has been reported previously for filamentous fungi, e.g., *P. chrysogenum* by PAFB [10], *Penicillium digitatum* by PdAfpB [51], or *P. expansum* by PeAfpA and PeAfpB [25]. Though still controversial, this observation could be explained with an additional function of the MPs, apart from their growth inhibitory activity, such as autophagy, nutrient recycling, and regulation of growth and development [10,14,52,53].

Furthermore, we investigated in this study the potential of PAFC as a bio-fungicide. It is the first time that the efficacy of a member of the BP-clade to protect tomato plant leaves from fungal infection was evaluated. The gray mold *B. cinerea* is a necrotrophic plant pathogen with the ability to infect numerous plant species, causing an estimated annual economic damage of one billion euros [54,55]. One of the hosts is the tomato plant (*Solanum lysopersicum*), an important and widely used agricultural crop. So far, infection control was managed by chemical fungicides [56], which, in itself, proves to be a problem due to residues in tomato fruits and the risk of toxicity for consumers [57,58]. Additionally, recent evidence showed that *B. cinerea* has already acquired resistance to many of the commonly used fungicides [59]. Apart from the effective reduction of tomato plant leaf destruction by mold-induced necrosis in the presence of PAFC, we also proved the tolerance of tomato leaves and *M. truncatula* seedlings to PAFC treatment. In-depth studies are currently in progress to further investigate the applicability of PAFC in agricultural and post-harvest settings to prevent and treat fungal plant infection and fruit decay.

Taken together, the antifungal spectrum, which includes human as well as plant pathogens, suggests PAFC to be a potential candidate for the development of new antifungal strategies applicable in the clinics and agriculture.

## 4. Materials and Methods

### 4.1. Microbial Strains and Growth Conditions

Fungal and bacterial strains used in this study are listed in Supplementary Table S3, and the composition of the media is described in Supplementary Table S4. Spores were generated by cultivating *P. chrysogenum* on *P. chrysogenum* minimal medium (*Pc*MM) agar plates at 25 °C and *A. fumigatus*, *A. nidulans*, and *A. niger* on solid complete medium (CM) at 37 °C. *N. crassa* was grown on Vogel’s agar plates at 37 °C under light. The dermatophytes *M. gypseum* and *T. rubrum* were cultivated on oatmeal agar at 30 °C. *B. cinerea* was cultivated on potato dextrose agar (PDA; Sigma-Aldrich, St. Louis, MO, USA) and incubated first at 30 °C for 48 h and then at 25 °C for 10 days. All fungal spores were routinely harvested and washed in spore buffer (0.9% NaCl (*w*/*v*), 0.01% Tween (*v*/*v*)) before use, except for the spores of dermatophytes, which were harvested in spore buffer and filtered through a funnel lined with cotton to remove hyphae before washing the spores. A single colony of *C. albicans* was removed from PDA, transferred to liquid 0.1 × PDB, and grown at 160 rpm and 30 °C. After centrifugation, the cells were washed in the same medium before experimental use. *E. coli* and *B. subtilis* were grown as a preculture in lysogeny broth (LB) medium until an OD_600nm_ of 0.3 was reached.

### 4.2. PAFC Production

We inoculated 200 mL of *Pc*MM with 2 × 10^8^ mL^−1^ spores of the PAFC-overexpressing strain *P. chrysogenum^OepafC^* [14], and PAFC was purified from the cell-free supernatant of a 96-h-old shaking culture (25 °C, 200 rpm), as previously described [14]. Isotopic ^15^N/^13^C labeling of recombinant PAFC for NMR analysis was achieved by replacing the carbon and nitrogen source in *Pc*MM with 1% ^13^C-glucose (*w*/*v*) (Euriso-Top, Saarbrücken, Germany) and 0.3% Na^15^NO_3_ (*w*/*v*) (Euriso-Top, Saarbrücken, Germany), respectively. The isotopic-labeled PAFC was produced and purified in the same way as the unlabeled MP. 

### 4.3. NMR Measurements, Signal Assignment, and Structure Calculations 

Lyophilized PAFC samples were dissolved in 20 mM acetate buffer (pH = 4.5, 5% D_2_O) in a concentration of 650 μM. For NMR signal assignments, the standard protocol applied for other ^15^N-/^13^C-isotope-labeled antifungal MPs [60,61] was used. Thus, HNCO-, HN(CA)CO-, HNCA-, HN(CO)CA-, HNCACB-, HNHA-, HBHA(CO)NH-, and HN(CO)CACB-type three-dimensional triple-resonance experiments were recorded to identify sequential connections through the protein backbone. The side-chain resonance assignments were completed with the help of HC(C)H COSY, HC(C)H-TOCSY, HCC(CO)NH, and (H)CCH-TOCSY experiments. In the case of aromatic protons, two-dimensional CB(CGCD)HD and CB(CGCDCE)HE spectra were used. For NOE peak assignment and structure calculation, ^15^N- and ^13^C-resolved three-dimensional NOESY as well as 2D ^1^H-^1^H NOESY spectra were acquired. The identification of H-bonds was accomplished using the HNCOGPHB3D Bruker pulse sequence experiment [34]. All spectra were recorded at 298 K using a Bruker NEO/Avance III 700 MHz spectrometer (Bruker, Billerica, MA, USA). Typical 90° pulses were 8.1, 12, and 32 μs for ^1^H, ^13^C, and ^15^N, respectively, and relaxation delays were in the 1.1–1.7 s range. Direct chemical shift referencing was performed for ^1^H using 2,2-dimethyl-2-sila-pentane-5-sulfonic acid (DSS), (Sigma-Aldrich, St. Louis, MO, USA) and an indirect one for ^15^N and ^13^C nuclei calculated from the gyromagnetic ratios. The spectra were processed using Topspin 3.1, and the resonances were identified and analyzed with CCPNmr Analysis 2.4.2 software [62]. For collecting backbone torsional angle restraints, the TALOS-N webserver was chosen [63,64]. The disulfide pattern was considered the same as in the case of BP, and these bonds were defined as covalent bond restraints for the structure calculation. NOE peak picking and assignment as well as the structure determination of PAFC were accomplished with the UNIO’10 platform [65,66], which is based on the ATNOS/CANDID algorithm and CYANA 2.1 software. For determining the initial protein fold, only NOE distance constraints were considered. The refinement of the three-dimensional structure was completed with disulfide bond pattern and backbone torsional angle restraints [64]. In the final structure calculation, 1421 NOEs were included in the optimization. As usual, 100 conformers were determined, and the 20 lowest-energy structures of them were selected to obtain the final structural ensemble. For the dynamical studies of PAFC, conventional ^15^N (70.966 MHz), relaxation experiments (T_1_, T_2_, and ^15^N-^1^H NOE) were also run on the Bruker NEO/Avance III 700 MHz spectrometer using the ^15^N-/^13^C-labeled PAFC sample at 298 K. ^15^N-{^1^H} NOEs were measured using Bruker’s hsqcnoef3gpsi pulse program. The recycle delay was 7 s in the reference experiment, and the same duration was applied for the pre-saturation ^1^H pulse train, which was a train of 120° proton pulses (10.8 μs), followed by 5 ms delays in the on-resonance experiment. ^15^N *T*_1_ and *T*_2_ relaxation times were measured using standard pulse sequences hsqct1etgpsi3d.2 and hsqct2etgpsi3d.2, with 3 s recycle delays. The ^15^N relaxation data were analyzed using the Lipari–Szabo method of Bruker Dynamics Center version 2.4.8 software (Bruker, Billerica, MA, USA). The global correlation time and the S^2^ order parameters for each aa residue were calculated applying the M2 model, which includes residue-specific order parameters and effective correlation times and one global correlation time of an isotropic rotor. To obtain more insight into the dynamical properties of PAFC, the same relaxation data were analyzed with the aid of reduced spectral density mapping [40] using in-house-written MATLAB code. CPMG-RD experiments [67,68] were recorded to disclose possible motions at the millisecond timescale. ^15^N CPMG-RD *T*_2_ relaxation dispersion experiments were carried out as a function of the applied B_1_ RF field. Bruker’s hsqcrexetf3gpsi3d pulse program was applied. The B_1_ field was changed in between the 0–1000 Hz range, in 13 steps. The recycle delay was 2 s.

### 4.4. ECD Spectroscopy

The secondary structure and thermal stability of PAFC were assessed using ECD spectroscopy. Spectra were recorded in the 195–260 nm wavelength range at a scan speed of 100 nm s^−1^ using a Jasco-J815 spectropolarimeter (JASCO Corporation, Tokyo, Japan). The protein sample was dissolved in H_2_O at a 0.1 mg mL^−1^ concentration and transferred to a 0.1-cm-path-length quartz cuvette for the following measurements. First, the ECD spectrum of the sample was recorded at 25 °C. The temperature was then gradually increased up to 95 °C at a rate of 1 °C min^−1^ using a Peltier thermo-electronic controller (TE Technology, Traverse City, MI, USA), while ellipticity data were recorded as a function of temperature at four wavelengths (195, 202, 215, and 229 nm) appointed by the extrema of the spectrum measured at 25 °C and characteristic wavelengths determined previously for related proteins [41,42,43,44]. The protein solution was equilibrated for 1 min at each temperature point before measurements were taken. The resultant melting curves were fitted with a symmetrical sigmoidal function whose inflexion points corresponded to the melting temperature (*T*_m_) of the protein structure. When the unfolding experiment reached its final temperature point at 95 °C, the ECD spectrum in the 195–260 nm range was recorded again, and then the sample was left to cool to 25 °C. The last spectrum acquisition was done at 25 °C, after 5 min equilibration. The reported spectra were accumulations of 10 scans, from which the spectrum of H_2_O was subtracted. Ellipticity data are given in mdeg units.

Contributions of spectral signatures to the ECD spectra, emerging from different secondary structural regions of the protein, were determined by the circular dichroism spectra secondary structure (CDSSTR) method [69]. Deconvolution data were then compared to values reported by DSSP analysis [36] of the NMR-derived structure reported here and the crystallographic structure of the homologous *P. brevicompactum* BP (pdb code 1UOY).

### 4.5. Synthesis of γ-Core Peptides PCγ15, PCγ17, and PCγ^C-terminal^

The peptides were prepared manually by solid-phase peptide synthesis, applying Fmoc chemistry and dicyclohexylcarbodiimide/N-hydroxybenzotriazole (DCC/HOBt) coupling with a three-fold excess of reagents. Rink amide resin was used as a solid support. As the last step of the synthesis, the N-terminal amino group of the peptide was acetylated on the solid phase. The peptide was cleaved simultaneously from the resin and the side chains deprotected with a 95:5:3 (*v*/*v*/*w*) trifluoroacetic acid (TFA) /water/dithiothreitol mixture at room temperature for 2.5 h. The crude peptide was purified by semi-preparative reversed-phase high-performance liquid chromatography (RP-HPLC) on a Phenomenex Luna 10 µm C18 column using the following eluent system: (A) 0.1% (*v*/*v*) TFA and (B) 80% (*v*/*v*) acetonitrile and 0.1% (*v*/*v*) TFA applying a linear gradient from 10% to 40% B (PCγ15 and PCγ17) or 0% to 30% B (PCγ^C-terminal^) in 60 min at a flow rate of 3.0 mL min^−1^. Purity was checked with a Phenomenex Luna 10 µm C18 100 Å column and a linear gradient from 28% to 43% B (PCγ15), from 22% to 37% B (PCγ17), or from 8% to 23% B (PCγ^C-terminal^) in 15 min (Supplementary Figure S6). The identity of the peptide was proven by electrospray ionization–mass spectrometry (ESI-MS).

### 4.6. Determination of Antimicrobial Activity 

The inhibitory concentration that reduces microbial growth by ≥90% (IC_90_) was determined using broth microdilution assays. To this end, 100 μL of fungal spores or yeast cells (10^4^ mL^−1^) were mixed with 100 μL of PAFC in 0.1 × PDB in increasing concentrations (dermatophytes: 0–20 μM; *Aspergillus* spp., *N. crassa*: 0–30 μM; *P. chysogenum*: 0–100 μM; *Candida*: 0–20 μM) in 96-well, flat-bottom microtiter plates (Thermo Scientific, Waltham, MA, USA). *E. coli* and *B. subtilis* precultures were grown in 0.1 × PDB until an OD_620nm_ of 0.3 was reached, and diluted to an OD_620nm_ of 0.1 and mixed with 100 μL of increasing PAFC concentrations (0–50 μM). The growth inhibition of the static cultures was evaluated by determining the optical density (OD_620nm_) with a multi-mode microplate reader (FLUOstar Omega, BMG Labtech, Ortenberg, Germany) after cultivation of *Aspergillus* spp. and *C. albicans* for 24 h at 30 °C. *N. crassa* was incubated for 16–30 h at 25 °C, and dermatophytes were cultivated for eight days at 30 °C. Bacterial growth was evaluated after 24 h of cultivation at 30 °C. All assays included a blank (culture broth without spores or cells) for background subtraction and respective untreated microorganisms, which served as a growth control representing 100% growth. All experiments were done in technical triplicates and repeated at least twice. Agar diffusion assays were performed to document the degree of hyphal extension inhibition by PAFC in *B. cinerea* surface culture. Solid culture medium (0.1 × PDA) was overlaid with 1 mL of 2 × 10^5^ conidia mL^−1^, and 100 μL of 0.25–1 mg mL^–1^ PAFC solution diluted in sterile ddH_2_O was filled into the wells, which had been punched into the solid medium. Sterile ddH_2_O was used as a negative inhibition control. The diameters of the inhibition zones that had formed around the wells were documented after incubation for 96 h at 25 °C. The experiment was repeated twice.

### 4.7. Toxicity Tests with Medicaco truncatula Seedlings

To exclude any potential toxic effects of PAFC during plant growth, an experiment with the plant seeds of *M. truncatula* A-17 was conducted [13]. In short, seeds were sterilized, placed on 1% (*w*/*v*) water agar (Kalys, Bernin, France), and germinated at 4 °C in the dark for three days. Seedlings with 3–4 mm root length were chosen for the experiment, and four seedlings per square Petri dish with vents (Greiner, Sigma-Aldrich, St. Louis, MO, USA) containing water agar were put next to each other, keeping a 20 mm distance from the top. The apical region of the primary root was treated for 10 days with daily doses of an aqueous 20 µL PAFC solution (1 mg mL^−1^) and incubated in a 60% humid plant growth chamber at 23 °C and 1200 lux illumination for the leaf region, while evolving roots were kept in the dark by covering the respective region of the dish with aluminum foil. The primary root length was measured in millimeters and the number of lateral roots counted at day 10 of incubation. Seedlings treated with ddH_2_O and 70% (*v*/*v*) ethanol were used as growth and death controls, respectively. The toxicity test was repeated at least twice.

### 4.8. Plant Protection Experiments

Tomato plant seeds (*Solanum lycopersicum* L. cv. Ailsa Craig) were germinated for three days at 27 °C under darkness, and then the seedlings were transferred to Perlite for 14 days and grown in a controlled environment (200 μmol m^−2^ s^−1^ photon flux density with 12/12 h light/dark period, a day/night temperature of 23 °C/20 °C, and a relative humidity of 55%–60% for 4 weeks) in hydroponic culture [70]. The plant protection experiments were conducted from 9:00 a.m. For the assessment of PAFC efficacy to protect against fungal infection, the pathogenicity test method described by [71,72] was adopted with slight modifications. Detached tomato plant leaves were positioned in Petri dishes containing three sterilized filter papers (Filters Fioroni, Ingré, France) wetted with sterile ddH_2_O. (i) For infection control, 10 µL of *B. cinerea* SZMC 21,472 conidial suspension (1 × 10^7^ conidia mL^−1^), (ii) for PAFC toxicity testing, 10 µL of 1 mg mL^−1^ PAFC, (iii) for plant protection investigation, 10 µL of *B. cinerea* conidial suspension (1 × 10^7^ conidia mL^−1^) containing PAFC in a concentration range of 0.25–1 µg mL^−1^, and (iv) for the un-infected control, 10 µL of 0.1 × PDB was dropped onto the abaxial leaf epidermis at three points between the later veins and left to dry on the surface at room temperature. Conidial suspensions and PAFC solutions were prepared in 0.1 × PDB for the tests. After these treatments, leaves were kept in a humid (60%) plant growth chamber for four days at 23 °C under photoperiodic day/night simulation (12/12 h with/without illumination at 1200 lux). Leaves left untreated were used as untreated controls. After the incubation period, the leaves were collected and the necrotic zone around the treatment points and the necrotic lesions were visualized by Evan’s blue staining. Briefly, the leaves were stained with 1% (*w*/*v*) Evan’s blue (Sigma-Aldrich, St Louis, MO, USA) for 10 min according to [73] and then rinsed with distilled water until they were fully decolorized. Then the chlorophyll content was eliminated by boiling in 96% (*v*/*v*) ethanol for 15 min. The leaves were stored in a glycerine:water:alcohol (4:4:2) solution and photographed with a Canon EOS 700D camera (Tokyo, Japan). Three leaves for each treatment were used in one experiment. The plant protection experiment was repeated twice.

## Figures and Tables

**Table 1:** Observed long-range hydrogen bonds in PAFC, identified in the hncogphb3d NMR spectrum [34].

Secondary Structure Elements	Residue with Amide Hydrogen	Residue with Carbonyl Oxygen
β1-β2	29Gly	34Gly
β1-β2	36Val	27Phe
β1-β2	27Phe	36Val
β2-β3	35Ile	47Gln
β2-β3	37Glu	44Thr
β2-β3	39Lys	42Lys
β2-β3	44Thr	37Glu
β2-β3	46Ile	35Ile
β2-β3	49Cys	33Thr
β4-β5	55Arg	63Arg
β4-β5	57Val	60Gly
β4-β5	63Arg	55Arg
N-terminal-β1	12Arg	28Cys
N-terminal-β1	28Cys	10Asp
N-terminal-β3	15Asn	43Trp
N-terminal-β3	43Trp	16Ser
loop1-β4	54Cys	32Arg
N-terminal region	13Arg	1Asp

**Table 2:** Estimation of secondary structural components of PAFC from ECD spectra compared to those obtained from NMR measurements.

Secondary Structural Elements/%	PAFC 25 °C	PAFC 95 °C	PAFC 25 °C after Heating	BP Crystallographic Structure (1UOY) ^§^	PAFC Solution NMR Structure (6TRM) ^§^
α- or 3_10_-helix	13	15	14	5	6
β-strand, extended	28	26	28	39	38
β- or γ-turns, bends	26	26	25	31	33
unordered	33	34	32	25	23

^§^ The fraction of secondary structural elements was determined by DSSP analysis [36] of the NMR-derived PAFC structure and the crystallographic structure of the homologous *P. brevicompactum* BP [24].

**Table 3:** PAFC inhibitory concentrations that reduce the growth of microorganisms by ≥90% (IC_90_) ^$^.

Microorganism	IC_90_	
	µM	μg mL^−1^
*Aspergillus fumigatus*	>30	199
*Aspergillus nidulans*	30	199
*Aspergillus niger*	>30	199
*Neurospora crassa*	15	99
*Penicillium chrysogenum*	50	331
*Microsporum gypseum*	7	46
*Trichophyton rubrum*	5	33
*Bacillus subtilis*	>50	331
*Escherichia coli*	>50	331

^$^ Broth microdilution assays were performed to determine the IC_90_. Microorganisms were grown in ten-fold-diluted potato dextrose broth (0.1 × PDB) for 24 h at 30 °C in static culture before measurement of the optical density at wavelength 620 nm (OD_620nm_). Exceptions: *N. crassa* was grown for 16–30 h at 25 °C, and *M.* *gypseum* and *T. rubrum* were grown for eight days at 30 °C. IC_90_ was defined as the PAFC concentration that inhibited growth by ≥90% compared to the untreated control, which represented 100% growth.

**Table 4:** Physicochemical characteristics and the fungal growth inhibitory potential (IC_90_) of the peptides PCγ15, PCγ17, and PCγ^C-terminal^ compared to the full-length MP PAFC ^§^.

Protein/Peptide	Molecular Mass (kDa)	Charge (pH 7.0)	GRAVY	IC_90_			
				*N. crassa*		*C. albicans*	
				μM	μg mL^−1^	μM	μg mL^−1^
PAFC	6.6	+0.6	−0.767	12.5	83	2.5	17
PCγ15	1.5	−0.2	+0.087	>100	66	>100	66
PCγ17	1.8	+1.1	−0.367	25	166	>100	66
PCγ^C-terminal^	0.7	+0.8	+0.038	>100	66	>100	66

^§^ The calculated molecular mass (kDa, ExPasy ProtParam tool), the predicted net charge (at pH 7, http://protcalc.sourceforge.net), and the GRAVY value (www.gravy-calculator.de) are indicated. For determination of IC_90_ values in 0.1 × PDB, *C. albicans* was grown for 24 h at 30 °C and *N. crassa* for 16–30 h at 25 °C. IC_90_ was defined as a protein/peptide concentration that inhibited growth by ≥90% compared to the untreated control, which represented 100% growth.

**Table 5:** Inhibition of *Botrytis cinerea* surface culture growth by PAFC ^§^.

PAFC (mg mL^−1^)	Diameter of Inhibition Zone (mm)
0.25	16.5 ± 0.71
0.5	17.0 ± 0.00
0.75	18.5 ± 0.71
1.0	20.0 ± 0.00

^§^*Botrytis cinerea* SZMC 21,472 was grown on 0.1 × PDA plates for 96 h at 25 °C.

## Data Availability

The data presented in this study are available in supplementary material.

## Supplementary Materials

Supplementary Materials can be found at https://www.mdpi.com/1422-0067/22/3/1183/s1.

## Abbreviations

Aa	Amino acid
BP	*Penicillium brevicompactum* bubble protein
BMRB	Biological magnetic resonance data bank
CDSSTR	Circular dichroism spectra secondary structure
CPMG-RD	Carr–Purcell–Meiboom–Gill relaxation dispersion
DSSP	Dictionary secondary structures of protein
DI	Disorder index
ECD	Electronic circular dichroism
fASA	Fractional associated solvent area
GRAVY	Grand average of hydropathy value
HI-HSQC	Heteronuclear in-phase single quantum coherence
IC_90_	Inhibitory concentration reducing microbial growth by ≥90%
kDa	kiloDalton
K	Kelvin
MP(s)	Miniprotein(s)
NFAP	*Neosartorya fischeri* antifungal protein
NMR	Nuclear magnetic resonance
NOE	Nuclear Overhauser effect
OD	Optical density
PAF	*Penicillium chrysogenum* antifungal protein
PdAfp	*Penicillium digitatum* antifungal protein
PeAfp	*Penicillium expansum* antifungal protein
pdb	Protein Data Bank
RCI	Random coil index
RMSD	Root-mean-square deviation
