# Supplementary material for: Solution Structure, Dynamics, and New Antifungal Aspects of the Cysteine-Rich Miniprotein PAFC
Source: Int J Mol Sci. 2021 Jan 25;22(3):1183. doi: 10.3390/ijms22031183 (PMC7865535; doi:10.3390/ijms22031183)
Supplement: Supplementary file 1 [file ijms-22-01183-s001.pdf]

Supplementary Material

Solution Structure, Dynamics, and New Antifungal Aspects of the Cysteine-Rich Miniprotein PAFC

András Czajlik[1,†], Jeanett Holzknecht[2,†], László Galgóczy[3,4], Liliána Tóth[3,4], Péter Poór[5], Attila Ördög[5], Györgyi Váradi[6], Alexander Kühbacher[2], Attila Borics[7], Gábor K. Tóth[6,8], Florentine Marx[2,*] and Gyula Batta[1,*]

1 Department of Organic Chemistry, Faculty of Science and Technology, University of Debrecen, H-4032 Debrecen, Hungary; czajlik.andras@science.unideb.hu (A.C.)
2 Institute of Molecular Biology, Biocenter, Medical University of Innsbruck, A-6020 Innsbruck, Austria; jeanett.holzknecht@i-med.ac.at (J.H.); alexander.kuehbacher@i-med.ac.at (A.K.)
3 Institute of Plant Biology, Biological Research Centre, Eötvös Loránd Research Network, H-6726 Szeged, Hungary; galgoczi.laszlo@brc.hu (L.G.); toth.liliana@brc.hu (L.T.)
4 Department of Biotechnology, Faculty of Science and Informatics, University of Szeged, H-6726 Szeged, Hungary
5 Department of Plant Biology, Faculty of Sciences and Informatics, University of Szeged, H-6726 Szeged, Hungary; poorpeti@bio.u-szeged.hu (P.P.); aordog@bio.u-szeged.hu (A.Ö.)
6 Department of Medical Chemistry, Faculty of Medicine, University of Szeged, H-6720 Szeged, Hungary; varadi.gyorgyi@med.u-szeged.hu (G.V.); toth.gabor@med.u-szeged.hu (G.K.T.)
7 Institute of Biochemistry, Biological Research Centre, Eötvös Loránd Research Network, H-6726 Szeged, Hungary; borics.attila@brc.hu
8 MTA-SZTE Biomimetic Systems Research Group, University of Szeged, Dóm tér 8, H-6720 Szeged, Hungary

† These authors share first authorship.
* Correspondence: florentine.marx@i-med.ac.at (F.M.); batta@unideb.hu (G.B.)

Keywords: *Penicillium chrysogenum;* antifungal protein PAFC; γ-core motif; solution structure; dynamics; nuclear magnetic resonance; plant protection

Supplementary Tables

Table S1. Statistics of PAFC structure determination.

**Number of NOE restraints:**	**1421**
intraresidual	237 (16.68%)
sequential	389 (27.38%)
medium-range	171 (12.03%)
long-range	624 (43.91%)

Number of TALOS dihedral angle constraints: **96**

**Ramachandran statistics for the ensemble:**	
Residues in favored regions	92.82%
Residues in allowed regions	5.56%
Residues in disallowed regions	1.61%

Table S2. Summary of ^{15}N relaxation experiments.
(The highest disorder index values were found for the next residues in decreasing order: **Arg32** = 0.326, **Gly51** = 0.235, **Thr33** = 0.128, **Gly34** = 0.116)

**Residue**	**T_1**	**T_2**	**NOE**	**S^2(M2)**
1Asp	-	-	-	-
2Thr	0.4774	0.1979	0.7856	0.845
3Cys	0.4637	0.1784	0.7442	0.884
4Gly	0.4831	0.1773	0.7726	0.910
5Gly	0.5308	0.1633	0.6563	0.813
6Gly	0.5309	0.2411	0.7290	0.697
7Tyr	0.4956	0.2222	0.7630	0.793
8Gly	0.4570	0.1223	0.7589	0.884
9Val	0.4527	0.2032	0.7271	0.761
10Asp	0.4011	0.1715	0.7617	0.901
11Gln	0.4262	0.1892	0.7537	0.863
12Arg	0.4469	0.1864	0.7484	0.887
13Arg	0.4576	0.1917	0.7959	0.874
14Thr	0.4481	0.1490	0.7990	0.933
15Asn	0.4456	0.2116	0.7681	0.649
16Ser	0.4560	0.1985	0.8162	0.850
17Pro	-	-	-	-
18Cys	0.4312	0.1978	0.7386	0.865
19Gln	0.4620	0.2043	0.7766	0.824
20Ala	0.4735	0.1278	0.7648	0.901
21Ser	0.4644	0.2201	0.7956	0.779
22Asn	0.4336	0.2004	0.7422	0.774
23Gly	0.4427	0.1242	0.7269	0.868
24Asp	0.4701	0.1493	0.7837	0.921
25Arg	0.4522	0.1928	0.7892	0.873

26His	0.4698	0.2173	0.7938	0.801
27Phe	0.4508	0.2083	0.7476	0.700
28Cys	0.4442	0.1995	0.7557	0.772
29Gly	0.4356	0.1493	0.7420	0.880
30Cys	0.4377	0.1862	0.7516	0.886
31Asp	0.4488	0.1837	0.7978	0.910
32Arg	0.4432	0.0383	0.7920	0.948
33Thr	0.4357	0.1112	0.7704	0.907
34Gly	0.4305	0.1211	0.8358	0.966
35Ile	0.4346	0.1994	0.7182	0.804
36Val	0.4212	0.1915	0.7246	0.857
37Glu	0.4373	0.2103	0.7308	0.683
38Cys	0.4621	0.2081	0.7438	0.707
39Lys	0.4537	0.2069	0.8065	0.829
40Gly	0.4668	0.2194	0.8007	0.770
41Gly	0.4293	0.2096	0.8250	0.651
42Lys	0.4120	0.1907	0.7294	0.865
43Trp	0.4382	0.1919	0.7583	0.839
44Thr	0.4668	0.1542	0.7008	0.847
45Glu	0.4372	0.1275	0.7942	0.927
46Ile	0.4257	0.1943	0.7331	0.855
47Gln	0.4383	0.2056	0.7438	0.652
48Asp	0.4517	0.1436	0.7385	0.878
49Cys	0.4522	0.2005	0.7538	0.768
50Gly	0.4424	0.2014	0.8306	0.899
51Gly	0.9194	0.2104	0.2460	0.389
52Ala	0.4826	0.2286	0.7252	0.585
53Ser	0.4714	0.2197	0.7848	0.795
54Cys	0.4023	0.1736	0.8141	0.945
55Arg	0.4128	0.1792	0.7911	0.921
56Gly	0.4577	0.2033	0.7817	0.866
57Val	0.4332	0.1957	0.7365	0.825
58Ser	0.4521	0.2030	0.8119	0.848
59Gln	0.4556	0.1981	0.7313	0.799
60Gly	0.4436	0.2103	0.7843	0.626
61Gly	0.4761	0.2249	0.6793	0.628
62Ala	0.4216	0.1839	0.7904	0.895
63Arg	0.4438	0.2040	0.7751	0.708
64Cys	0.4656	0.2149	0.7028	0.690

Table S3. Fungal and bacterial strains used in this study.

Organism	Strain	Source§
Aspergillus fumigatus	44645	ATCC
Aspergillus nidulans	A4	FGSC
Aspergillus niger	12049	CBS
Bacillus subtilis	6633	ATCC
Botrytis cinerea	21427	SZMC
Candida albicans	5982	CBS
Escherichia coli	DH5α	NEB
Microsporum gypseum	24102	ATCC
Neurospora crassa	4200	FGSC
Penicillium chrysogenum Q176	10002	ATCC
P. chrysogenum^{OEpafC}	PAFC overproducing strain	[1]
Trichophyton rubrum	28188	ATCC

§ATCC (LGC Standards, Wesel, Germany), CBS (Westerdijk Institute, Utrecht, The Netherlands), FGSC (Kansas State University, Manhattan, KS, US), NEB (Ipswich, MA, USA), SZMC (Szeged Microbiological Collection, University of Szeged, Szeged, Hungary).

Table S4. Composition of media and solutions used in this study.

**Medium**	**Abbreviation**	**Composition/Company**[§]
Aspergillus nidulans complete medium	CM	2% salt solution CM, 0.1% trace elements CM (*v/v*), 2% D(+)- glucose, 0.2% bacteriological peptone, 0.1% yeast extract, 0.1% NZ-Amine (*w/v*), pH 6.5
Salt solution CM	-	2.6 % KCl, 2.6% $MgSO_4 \times 7\ H_2O$, 7.6% KH_2PO_4 (*w/v*), 0.2% chloroform (*v/v*)
Trace elements solution CM	-	1.3% $ZnSO_4 \times 7\ H_2O$, 0.07% $CuSO_4 \times 5\ H_2O$, 0.1% $MnSO_4 \times H_2O$, 0.006% $Na_2B_4O_7 \times 10\ H_2O$, 0.13% $Na_2MoO_4 \times 2\ H_2O$, 0.23% $FeSO_4 \times 7\ H_2O$ (*w/v*)
Lysogeny broth medium	LB medium	1% NaCl, 1% bacteriological peptone, 0.5% yeast extract (*w/v*)
Oatmeal agar	-	1% powdered oatmeal, 0.15% KH_2PO_4, 0.1% $NaNO_3$, 0.1% $MgSO_4$, 2% agar (*w/v*)
Penicillium chrysogenum minimal medium[$]	*Pc*MM	0.3% $NaNO_3$, 0.05% $MgSO_4 \times 7\ H_2O$, 0.05% KCl, 0.005% $FeSO_4 \times 7\ H_2O$, 2% D(+)-sucrose (w/v), 2.5% 1 M KPO_4-buffer (pH 5.8), 0.1% trace elements *Pc*MM (*v/v*)
Trace element solution *Pc*MM	-	0.1% $FeSO_4 \times 7\ H_2O$, 0.9% $ZnSO_4 \times 7\ H_2O$, 0.04% $CuSO_4 \times 5\ H_2O$, 0.01% $MnSO_4 \times H_2O$, 0.01% H_3BO_3, 0.01% $Na_2MoO_4 \times 2\ H_2O$ (*w/v*)
Potato dextrose agar	PDA	PDB, 2% agar (*w/v*)
Potato dextrose broth	PDB	Sigma-Aldrich, St Louis, MO, USA
Vogel's medium	-	2% salt solution Vogel's medium (v/v), 2% D(+)-sucrose (*w/v*)
Salt solution Vogel's medium	-	15% sodium citrate, 25% KH_2PO_4, 10% NH_4NO_3, 1% $MgSO_4 \times 7\ H_2O$, 0.1% $CaCl_2$ (*w/v*), 0.1% trace elements Vogel's medium, 0.05% biotin (*v/v*)
Trace element solution Vogel's medium	-	5% citric acid × H_2O, 5% $ZnSO_4 \times 7\ H_2O$, 0.97% $FeSO_4 \times 7\ H_2O$, 0.25% $CuSO_4 \times 5\ H_2O$, 0.05% $MnSO_4 \times H_2O$, 0.05% H_3BO_3, 0.05% $Na_2MoO_4 \times 2\ H_2O$ (*w/v*)

[§]Percent values are given as weight per volume (*w/v*) for solids and volume per volume (*v/v*) for solutions. [$]For NMR-based analyses, isotopic labeling of PAFC was performed in *Pc*MM by replacing 0.3% $NaNO_3$ with 0.3% $Na^{15}NO_3$ and 2% sucrose with 1% ^{13}C-glucose (Euriso-Top, Saarbrücken, Germany).

Supplementary Figures

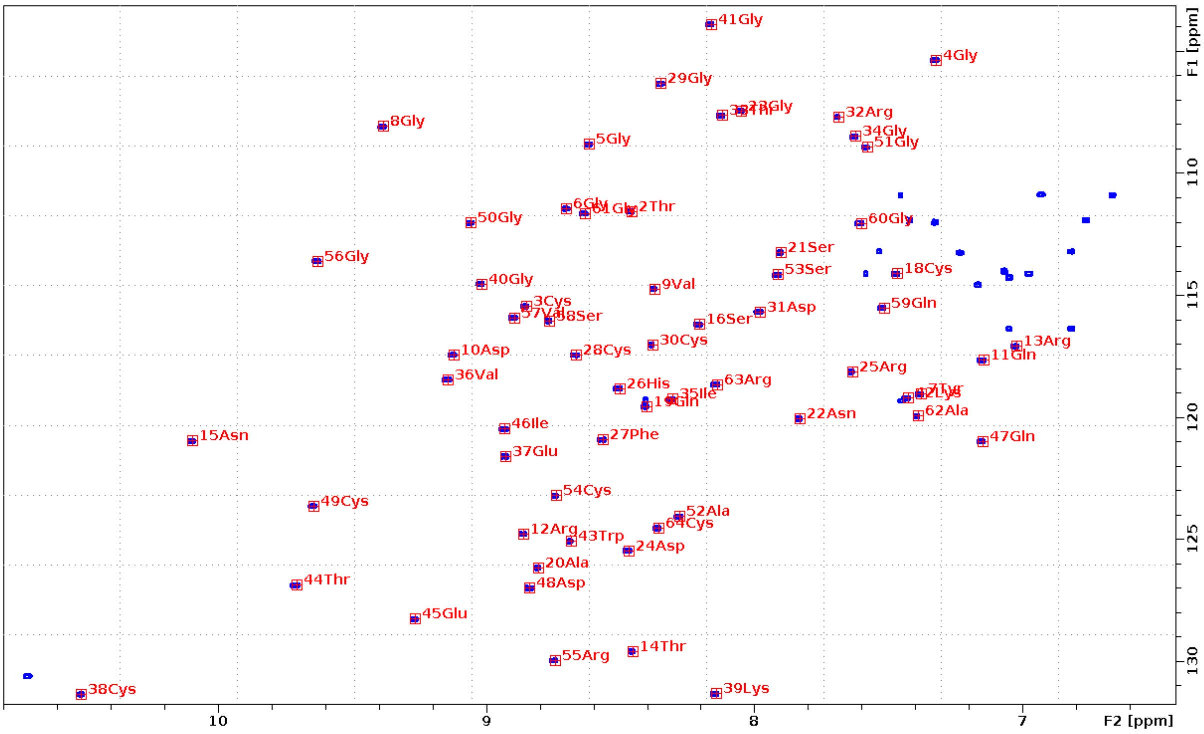

Figure S1. ^{15}N-HSQC spectrum of $^{13}C/^{15}N$ labeled PAFC, measured at 298K temperature. Chemical shift assignment data are deposited to BMRB, entry no. 34468. Only the main chain amide NH groups are labeled.

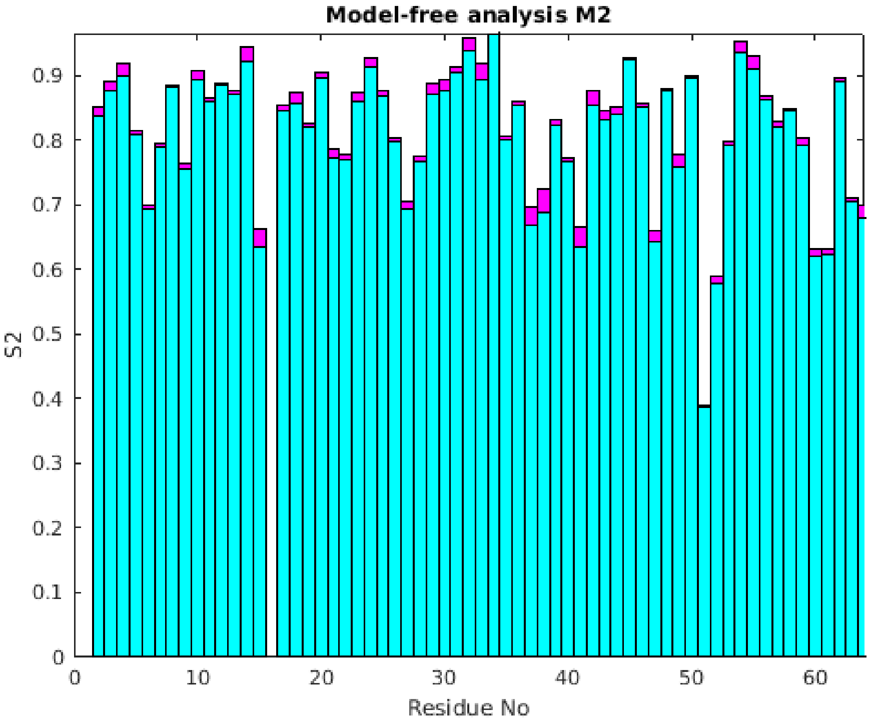

Figure S2. Model-free dynamics analysis of PAFC from ^{15}N relaxation parameters T_1, T_2 and ^{15}N-{^1H} NOE measured at 298K. Cyan bars represent the S^2 values, while the pink bars on the top show the error range. The numbers are identical with those shown in Table S2. For folded proteins S^2 values are typically in the 0.7–0.9 range, lower values indicate higher mobility in the ps–ns timescale. Validity of the model-free approach depends on the separation (two orders of magnitude) of the time scales of fast local, and slow global motions.

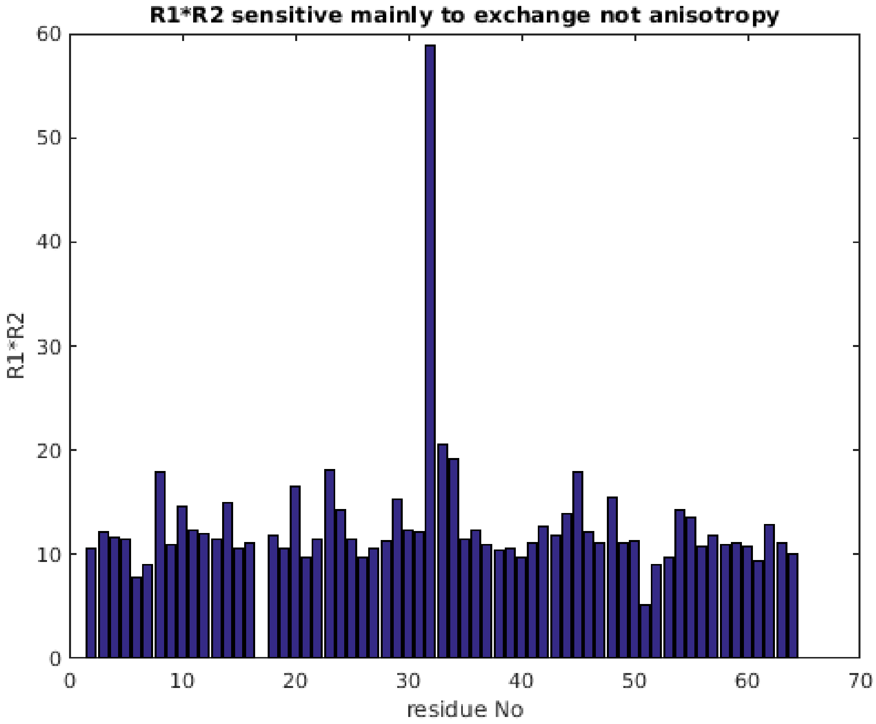

Figure S3. Raw R_1*R_2 ^{15}N-relaxation rates data shown here are sensitive to exchange processes [2] in PAFC. Relaxation rates are calculated as follows: R_1=1/T_1, R_2=1/T_2.

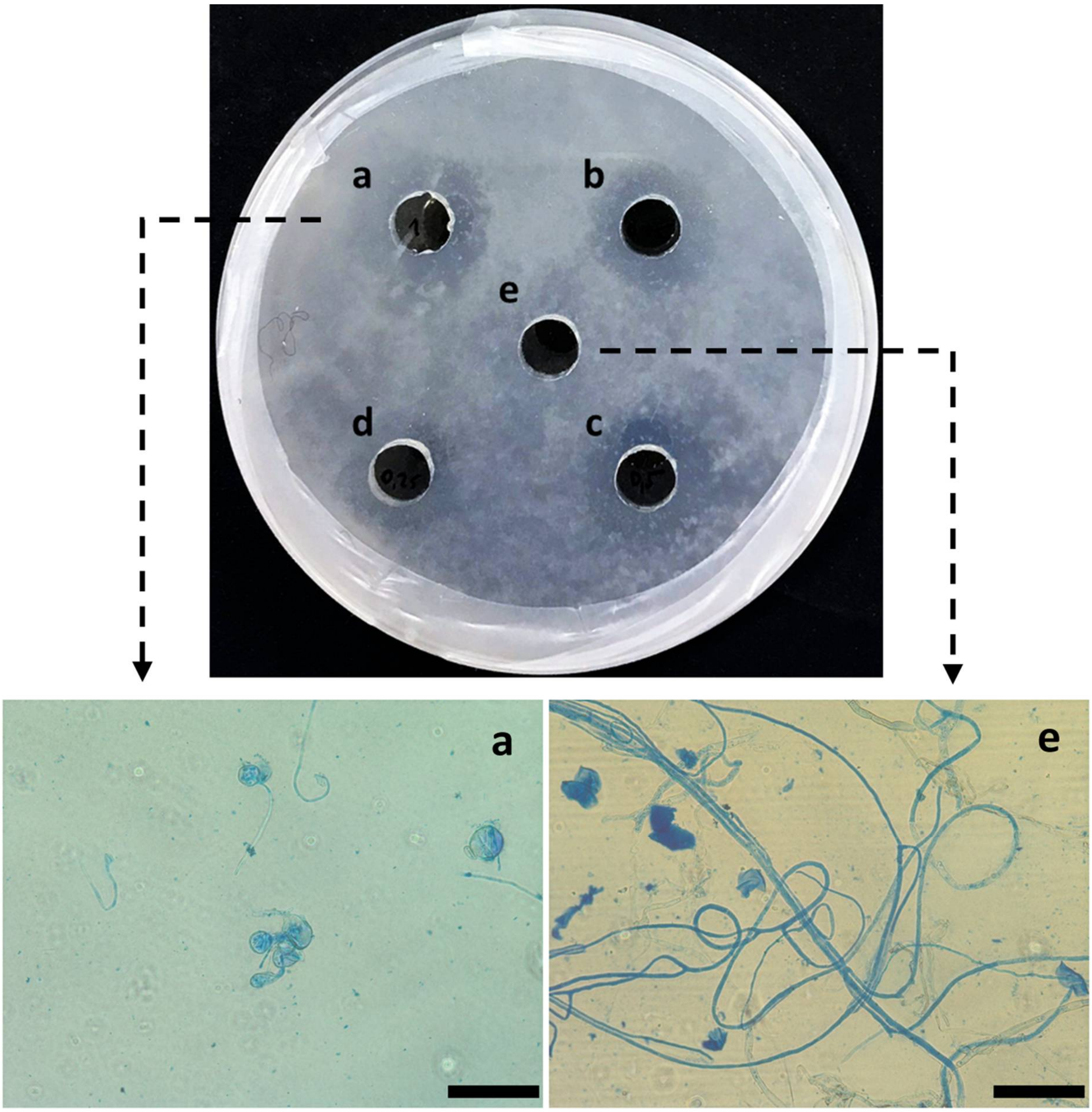

Figure S4. Inhibition of *Botrytis cinerea* SZMC 21472 growth by PAFC on 0.1 × PDB agar after incubation for 96 h at 25 °C. **Upper panel:** The wells contained 100 µL PAFC solution, diluted in ddH_2O at concentrations of **(a)** 1.0 mg mL^{-1}, **(b)** 0.75 mg mL^{-1}, **(c)** 0.5 µg mL^{-1} and **(d)** 0.25 mg mL^{-1}. **(e)** 100 µL ddH_2O was used as negative control. **Lower panel:** Microscopic imaging of hyphal growth within the zones around the wells containing **(a)** 1 mg mL^{-1} PAFC, and **(e)** ddH_2O. Lactophenol blue solution (Sigma-Aldrich, St. Louis, MO, USA) was applied to stain the fungal cells. Scale bars, 50 µm.

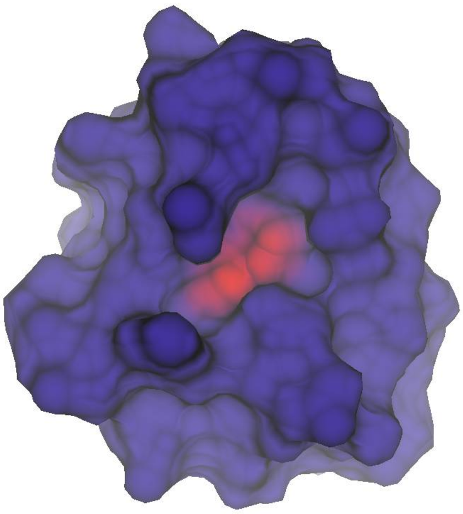

Figure S5. Visualization of cavity A in PAFC (6TRM) (residues involved: 1, 2, 7, 8, 9, 12, 13, 16, 17, 18, 19, 21, 22, 25, 27, 43, 58, 59) that is analogous to the cavity found in BP (1UOY).

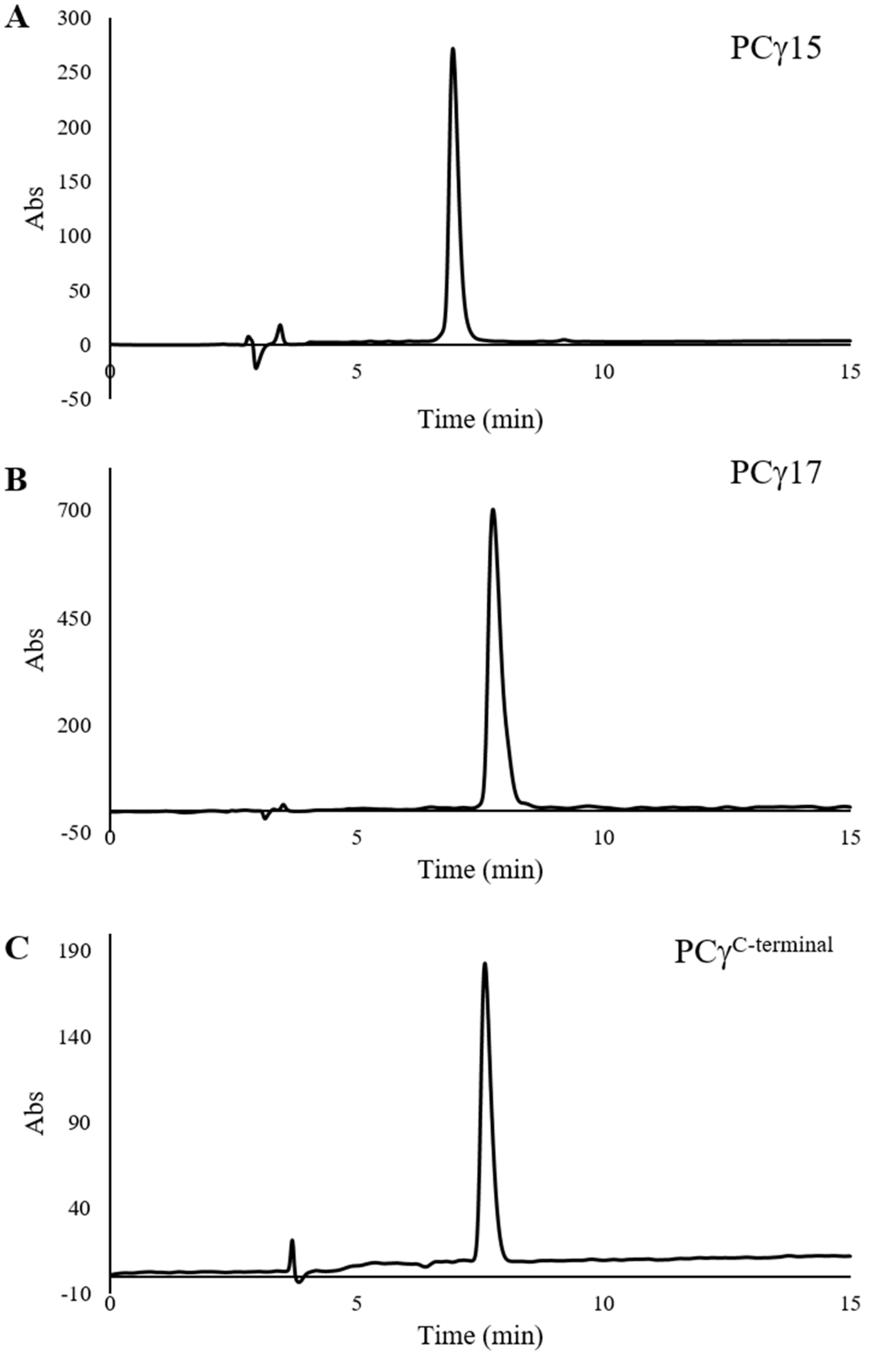

Figure S6. Reversed phase-high performance liquid chromatography profile of **(A)** PCγ15, **(B)** PCγ17, and **(C)** PCγ$^{C\text{-terminal}}$. A linear gradient was applied from 28 to 43% (**A**), 22 to 37% (**B**), and 8 to 23% (**C**) (*v/v*) solvent [B] in 15 min. Solvent system: [A] 0.1% (*v/v*) TFA; [B] 80% (*v/v*) ACN, 0.1% (*v/v*) TFA.

Supplementary Materials and Methods

We applied two recent *in silico* approaches that can be used for cavity search and characterization: CavityPlus [3] and CASTp 3.0 [4]. Both methods predict that PAFC has two cavities. CASTp program predicted smaller volume for cavity A (41 Å^3) than for cavity B (155 Å^3) (residues involved: 3, 12, 13, 14, 28, 29, 30, 31, 33, 34, 35, 36, 44, 45, 48). The volume underestimation of cavity A may happen because only a fraction of the irregular shape is recognized. CavityPlus code does not give explicit volumes but gives the visible size and pdb atom coordinates. Importantly, the residue numbers belonging to the same A cavities in PAFC (1, 2, 7, 8, 9, 12, 13, 16, 17, 18, 19, 21, 22, 25, 27, 43, 58, 59) and BP (1, 2, 12, 16, 17, 18, 19, 20, 22, 25, 27, 41, 43, 58, 59) seem to be similar.

Supplementary References

1. Holzknecht, J.; Kühbacher, A.; Papp, C.; Farkas, A.; Váradi, G.; Marcos, J.F.; Manzanares, P.; Tóth, G.K.; Galgóczy, L.; Marx, F. The *Penicillium chrysogenum* Q176 antimicrobial protein PAFC effectively inhibits the growth of the opportunistic human pathogen *Candida albicans*. *Journal of Fungi* **2020**, *6*, doi:10.3390/jof6030141.
2. Kneller, J.M.; Lu, M.; Bracken, C. An effective method for the discrimination of motional anisotropy and chemical exchange. *Journal of the American Chemical Society* **2002**, *124*, 1852-1853, doi:10.1021/ja017461k.
3. Xu, Y.J.; Wang, S.W.; Hu, Q.W.; Gao, S.S.; Ma, X.M.; Zhang, W.L.; Shen, Y.H.; Chen, F.J.; Lai, L.H.; Pei, J.F. CavityPlus: a web server for protein cavity detection with pharmacophore modelling, allosteric site identification and covalent ligand binding ability prediction. *Nucleic Acids Research* **2018**, *46*, W374-W379, doi:10.1093/nar/gky380.
4. Tian, W.; Chen, C.; Lei, X.; Zhao, J.L.; Liang, J. CASTp 3.0: computed atlas of surface topography of proteins. *Nucleic Acids Research* **2018**, *46*, W363-W367, doi:10.1093/nar/gky473.
